# Mechanistic target of rapamycin signaling in human nervous system development and disease

**DOI:** 10.3389/fnmol.2022.1005631

**Published:** 2022-09-26

**Authors:** Marie Girodengo, Sila K. Ultanir, Joseph M. Bateman

**Affiliations:** ^1^Kinases and Brain Development Lab, The Francis Crick Institute, London, United Kingdom; ^2^King’s College London, Maurice Wohl Clinical Neuroscience Institute, London, United Kingdom

**Keywords:** mTOR, neuron, organoid, mTORopathy, cortex, tuberous sclerosis

## Abstract

Mechanistic target of rapamycin (mTOR) is a highly conserved serine/threonine kinase that regulates fundamental cellular processes including growth control, autophagy and metabolism. mTOR has key functions in nervous system development and mis-regulation of mTOR signaling causes aberrant neurodevelopment and neurological diseases, collectively called mTORopathies. In this mini review we discuss recent studies that have deepened our understanding of the key roles of the mTOR pathway in human nervous system development and disease. Recent advances in single-cell transcriptomics have been exploited to reveal specific roles for mTOR signaling in human cortical development that may have contributed to the evolutionary divergence from our primate ancestors. Cerebral organoid technology has been utilized to show that mTOR signaling is active in and regulates outer radial glial cells (RGCs), a population of neural stem cells that distinguish the human developing cortex. mTOR signaling has a well-established role in hamartoma syndromes such as tuberous sclerosis complex (TSC) and other mTORopathies. New ultra-sensitive techniques for identification of somatic mTOR pathway mutations have shed light on the neurodevelopmental origin and phenotypic heterogeneity seen in mTORopathy patients. These emerging studies suggest that mTOR signaling may facilitate developmental processes specific to human cortical development but also, when mis-regulated, cause cortical malformations and neurological disease.

## Introduction

**FIGURE 1:**
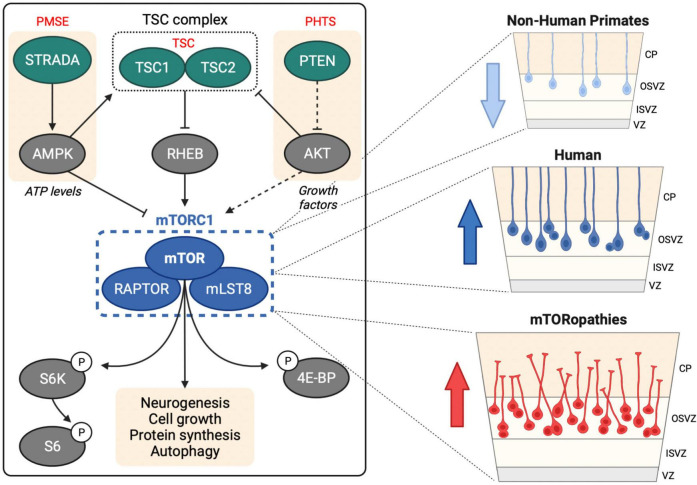
mTOR signaling in human neurogenesis. Left hand panel shows upstream regulation of mTORC1 by the TSC complex, STRADA and PTEN, mutations in which cause TSC, PMSE and PHTS, respectively. mTORC1 regulates several downstream processes through direct and indirect targets including S6 kinase (S6K), ribosomal protein S6 (S6) and eukaryotic translation initiation factor 4E (eIF4E)-binding protein (4E-BP). Right hand panel shows oRG cells in the human OSVZ, which are less prevalent in non-human primates. Activation of mTOR signaling in oRG may contribute to MCD in mTORopathies.

Mechanistic target of rapamycin (mTOR) is a large serine/threonine kinase that constitutes the catalytic subunit of two signaling complexes: mTOR complex 1 (mTORC1) and complex 2 (mTORC2) (Saxton and Sabatini, 2017). mTOR is highly conserved in eukaryotes and was first identified in *S. cerevisiae* (Cafferkey et al., 1993; Kunz et al., 1993; Helliwell et al., 1994). mTORC1 senses and integrates diverse cues from cellular nutrients, growth factors, insulin and stress to regulate cell growth, protein synthesis, autophagy and lipogenesis (Figure 1; Liu and Sabatini, 2020).

mTOR is also a key regulator of neurogenesis in both invertebrates and mammals (Lipton and Sahin, 2014; Tee et al., 2016). An early genetic screen for genes causing defects in mouse forebrain development showed that loss of mTOR caused failure of the telencephalic vesicles to form (Hentges et al., 1999). Further support for a conserved role for the mTOR pathway in neurogenesis came from studies of *Drosophila* photoreceptor differentiation (Bateman and McNeill, 2004, 2006; McNeill et al., 2008; Avet-Rochex et al., 2014; Maierbrugger et al., 2020). Since these pioneering studies, mTOR signaling has been shown to regulate many aspects of nervous system development, and to be involved in numerous neurological disorders (LiCausi and Hartman, 2018; Polchi et al., 2018).

mTORC1 is a major regulator of brain size. Deletion of *mTOR* in mouse neural stem cells (NSCs) from embryonic day (E) 10.5 causes microcephaly and a reduction in cortical thickness, due to decreased proliferation and cell size (Ka et al., 2014). Deletion of the mTORC1-specific component *Raptor* at the same stage also results in microcephaly, with reduced cell proliferation and growth (Cloetta et al., 2013). Conversely, activation of mTORC1 signaling through conditional deletion of *tuberous sclerosis complex 1* (*Tsc1*) or *2* (*Tsc2*), which encode upstream mTORC1 inhibitors, caused megalencephaly and increased cortical thickness (Magri et al., 2013). Activation of mTORC1 using an activated form of Ras-homolog enriched in brain (Rheb) at E15 also results in increased neuronal cell size (Nguyen et al., 2019).

Genetic modulation of mTORC1 signaling has shown that its ability to regulate brain size may result from effects on neural cell fate rather than proliferation. Decreasing mTORC1 activity via knockdown (KD) of *Rheb* prevents differentiation of mouse NSCs into intermediate progenitors, thus reducing neuronal production. Conversely, hyperactivating mTORC1 using constitutively active *Rheb* (*Rheb^CA^*) causes accelerated differentiation of NSCs into neural progenitor cells (NPCs) (Hartman et al., 2013). Similarly, mTORC1 activation through KD of the upstream inhibitor *RTP801/REDD1* at E17 triggers precocious and increased rate of differentiation of rat NPCs into neurons (Malagelada et al., 2011).

Our knowledge of mTORC1’s role in nervous system development comes primarily from rodent studies, leaving many questions on human neurodevelopment and human diseases unanswered. Recent technological advances, including increasingly powerful high-throughput omics technologies, and the progress made in the generation of human-derived induced pluripotent stem cells (hiPSCs) and human-derived cerebral organoids (hCOs), have offered new ways to investigate the developing human brain (Giandomenico and Lancaster, 2017). In this mini review, we focus on recent studies that have provided novel insights into the role of mTORC1 in human neurodevelopment and associated disorders. For a more comprehensive overview on mTOR signaling in neurodevelopment and disease we recommend other recent reviews (Lipton and Sahin, 2014; Tee et al., 2016; Feliciano, 2020; Nguyen and Bordey, 2021).

## Recent progress on the role of mechanistic target of rapamycin signaling in human neurodevelopment

Humans diverged from chimpanzees, our closest extant primate relative, between ∼9.3 and 6.5 million years ago, toward the end of the Miocene (Almécija et al., 2021). One of the most striking characteristics of human brain evolution is the expansion in surface area of the neocortex (Herculano-Houzel, 2009). Our understanding of cortical development comes mostly from rodent studies but the rodent neocortex is non-folded (lissencephalic), compared to the richly folded gyrencephalic primate neocortex. Even compared to chimpanzees, the human neocortex has a 10-fold increase in surface area which may facilitate higher cognitive function (Rakic, 1995).

Cortical neurogenesis begins when neuroepithelial cells lining the ventricular walls transform into radial glial cells (RGCs), which divide asymmetrically producing intermediate progenitor cells (IPCs) within the subventricular zone (SVZ) (Taverna et al., 2014). RGC processes contact the ventricular and pial surfaces and provide a scaffold for IPCs and neuroblasts as they migrate toward the cortical plate (Franco and Muller, 2013). In primates the SVZ is expanded into inner (ISVZ) and outer (OSVZ) regions. The OSVZ contains radial glia-like cells (oRG cells) that have a basal fiber directed toward the pia but lack an apical projecting fiber (Lui et al., 2011). oRG cells differentiate from ventricular radial glia (vRG), which also produce truncated radial glia (tRG) that remain in the VZ (Nowakowski et al., 2016). oRG cells are highly proliferative and have an extended period of transit amplification, which may contribute to the evolutionary expansion of the neocortex (Reillo et al., 2011; Nonaka-Kinoshita et al., 2013; Pollen et al., 2015).

Single-cell RNA-sequencing (scRNA-seq) technology has revolutionized our understanding of brain complexity and has provided strong evidence that mTOR signaling is robustly active in oRG cells (Figure 1). scRNA-seq of primary human telencephalon samples during the early stages of cortical development identified conserved gene networks contributing to cortical neuron diversity (Nowakowski et al., 2017). This approach provided the first evidence of a role for mTOR signaling in human neurogenesis: compared to vRGs, tRGs and IPCs, human oRG cells had enriched expression of mTOR pathway components, including Rheb, mTOR and Raptor, and also exhibited increased phospho-ribosomal protein S6 (P-S6) expression, a readout of mTORC1 activity.

Elucidating the neurogenic processes that have driven human brain development since our divergence from recent ancestors is limited by the inaccessibility of primary brain tissue from great apes. Such studies are now possible with the development of cerebral organoid technologies. Combined with scRNA-seq, cerebral organoids have enabled comparative brain anatomy with single-cell resolution (Fernandes et al., 2021). A recent ground-breaking study revealed that mTOR signaling may have played an important evolutionary role in human neocortical development. Analysis of 48 primary embryonic human (10–22 weeks post conception) and 6 embryonic macaque (9–17 weeks post conception) cortex tissue samples with 56 cerebral organoids (5–15 weeks old) derived from human or chimpanzee iPSCs enabled identification of gene regulatory networks that contribute to human brain development and evolution (Pollen et al., 2019). Cross comparison of primary tissues and organoids identified 261 genes with human-specific expression patterns in cortical development and highlighted mTOR signaling. Phosphorylation of the mTORC1 target S6 was significantly increased in the OSVZ in human vs. macaque primary tissue and in RGCs in human vs. chimpanzee cerebral organoids. Moreover, activation of mTOR signaling in macaque cortical progenitors increased P-S6 levels, while knockdown of upstream receptor tyrosine kinases in organotypic slice culture of primary human samples caused inhibition of mTOR signaling in the OSVZ. This study provides compelling evidence that mTOR signaling may have contributed to the evolution of human cortical neurogenesis (Figure 1).

Following on from the study by Pollen et al. (2019), the same group used human organotypic cultures and cerebral organoids to test the functional requirement of mTOR signaling in oRG cells. Hyperactivation or inhibition of mTOR signaling for 6 days in organotypic cultures disrupted the OSVZ radial glial scaffold, reducing the length and altering the orientation of oRG basal processes (Andrews et al., 2020). Similar truncated and disoriented oRG cells were observed with long-term modulation (for 5 weeks) of mTOR signaling in cerebral organoids. Moreover, in live imaging experiments rapamycin treatment perturbed oRG migration and reduced the length of the primary fiber. Surprisingly, however, mTOR signaling did not affect oRG proliferation, either in dissociated primary human cortical progenitors or cerebral organoids. Overall, this study shows that mTOR signaling is required for maintenance of correct oRG morphology and migration (Figure 1).

## New insights into the neurodevelopmental origin and heterogeneity of mTORopathies

Hyperactivation of mTORC1 signaling during neurodevelopment results in a group of neurological disorders known as mTORopathies. mTORopathies share two major symptoms: Malformation of cortical development (MCD) and intractable epilepsy (Subramanian et al., 2019; Moloney et al., 2021). Examples of mTORopathies include tuberous sclerosis complex (TSC), hemimegalencephaly (HMEG), focal cortical dysplasia type II (FCDII), and polyhydramnios, megalencephaly and symptomatic epilepsy syndrome (PMSE). The mechanisms by which mTORC1 hyperactivity causes these syndromes remain poorly understood. hiPSCs and hCOs are providing new ways to model and gain insight into the development of mTORopathies. In addition, analyses of somatic mTORC1-activating mutations in patients with epilepsy and MCD are deepening our understanding of the phenotypic heterogeneity seen in mTORopathies.

PMSE, also known as Pretzel syndrome, is caused by homozygous loss-of-function germline mutations in the *STE20-related kinase adaptor alpha* (*STRADA*) gene (Figure 1). STRADA is an upstream inhibitor of mTORC1, and individuals with PMSE typically suffer from early-onset intractable epilepsy, neurocognitive delay and MCDs including megalencephaly or cerebral ventriculomegaly. Mouse models of PMSE (STRADA KD or KO animals) recapitulate several of these disease features including abnormal cortical lamination, ventriculomegaly and enhanced mTORC1 activity (Orlova et al., 2010; Parker et al., 2013; Patino and Parent, 2014; Dang et al., 2020).

Recent studies have used hiPSCs and hCOs to understand the effect of PMSE-causing germline mutations on human development. Cortical-like excitatory neurons originating from patient-derived hiPSCs exhibited increased mTORC1 signaling and neuronal cytomegaly (Dang et al., 2021). hCOs grown from patient-derived hiPSCs showed various PMSE-related anomalies throughout their development, including increased mTORC1 signaling, abnormal cortical lamination, delayed early (rosette-stage) neurogenesis, maintenance of NSC identity, increased NSC proliferation, disrupted apical-basal polarity, atypical primary cilia architecture, and increased oRG pool (Dang et al., 2021). These observations suggest that megalencephaly in PMSE might be caused by a combination of delayed neurogenesis, maintenance of NSC fate, increased NSC proliferation, and increased numbers of oRG cells, ultimately resulting in an increased NSC pool at the beginning of corticogenesis. Heterozygous germline *phosphatase and tensin homolog* (*PTEN*) mutations cause PTEN hamartoma tumor syndrome (PHTS) (Figure 1), an mTORopathy characterized by macrocephaly and cognitive impairments (Pilarski, 2019). Interestingly, transient delays in neurogenesis, increased NSC proliferation, surface expansion and folding were also observed in hCOs with a targeted deletion of *PTEN* (Li et al., 2017b). These two studies together suggest that hCOs are well-suited to modeling mTORopathies.

hCOs have also been recently used to identify a potential new mTORopathy. *RAB39b* encodes a small GTPase, mutations in which are associated with X-linked macrocephaly, autism spectrum disorder (ASD), intellectual disability and early onset Parkinsonism (Giannandrea et al., 2010; Wilson et al., 2014). Using CRISPR-targeted *RAB39b* mutated hiPSCs, Zhang et al. (2020) generated hCOs lacking RAB39b expression. RAB39b mutant hCOs had increased NPC proliferation and delayed differentiation, resulting in enlarged organoid size and increased neuron production. RAB39b was then shown to physically interact with both catalytic and regulatory subunits of phosphatidylinositol 3-kinase (PI3K), and RAB39b mutant oRG cells had increased P-AKT and P-S6 expression, indicating PI3K-AKT-mTOR pathway activation. Moreover, genetic or pharmacological inhibition of AKT rescued the P-S6 expression and the increased size of RAB39b mutants hCOs. This study again highlights the relevance of mTOR signaling to MCD and suggests that *RAB39b* deficiency may be classed as a new mTORopathy.

**FIGURE 2:**
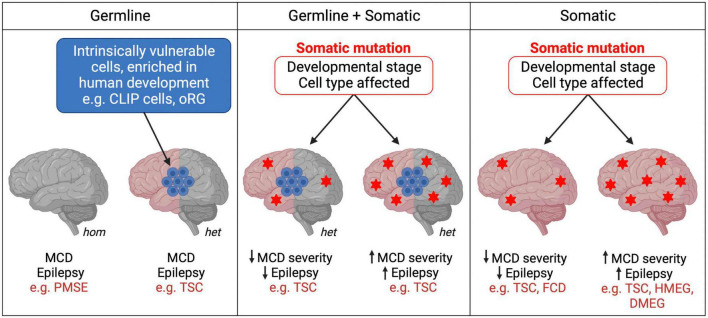
Germline and somatic mutations in mTORopathies. mTORopathies can arise from homozygous (hom) or heterozygous (het) germline mutations. In cell populations intrinsically vulnerable to mTORC1 hyperactivity during human neurodevelopment, heterozygous mutations could be sufficient to result in MCD (left panel). When somatic events follow a heterozygous germline mutation (e.g., second hits) (middle panel), or when they alone cause mTORopathies (right panel), the developmental stage and subset of cells affected might determine disease severity. Heterozygous *Tsc1/2* mutations (left panel) may also be sufficient to cause cellular changes, perhaps at later stages of neuronal development and maturation, as reported in hiPSC models, which contribute to the neurological manifestations of TSC.

TSC is the original mTORopathy, characterized by the formation of benign tumors (hamartomas) in multiple organs including the brain (Orlova and Crino, 2010). TSC patients present with various focal cortical lesions: tubers, tumors known as subependymal nodules (SENs) and subependymal giant cell astrocytomas (SEGAs), and cortical dysplasia. They also display neurological manifestations including treatment-resistant epilepsy, intellectual disability and ASD. TSC is caused by loss-of-function mutations in the mTOR pathway inhibitors *Tsc1* or *Tsc2*. The inheritance of TSC is autosomal dominant and can be caused by germline mosaicism, although most cases result from *de novo* mutations during early development (Rose et al., 1999; Orlova and Crino, 2010). Interestingly, hiPSC models have shown that neurons with heterozygous *Tsc1/2* mutations exhibit atypical morphology, defects in differentiation and axon guidance, and altered excitability and synaptic function (Li et al., 2017a; Sundberg et al., 2018; Zucco et al., 2018; Nadadhur et al., 2019; Catlett et al., 2021; Hisatsune et al., 2021). Homozygous loss of *Tsc1/2* in hiPSC-derived neurons generally enhances these phenotypes (Sundberg et al., 2018; Winden et al., 2019; Martin et al., 2020; Catlett et al., 2021; Hisatsune et al., 2021). Hamartomas are potentially caused by “second-hit” somatic mutations in *Tsc1* or *Tsc2* resulting in loss of heterozygosity (LOH) (Consortium, 1993; Sepp et al., 1996; van Slegtenhorst et al., 1997). The developmental stage and subset of cells affected by the second hit are proposed to determine the location and severity of the cortical lesions in TSC patients (Sepp et al., 1996; Martin et al., 2017; Figure 2). This second hit hypothesis is supported by an elegant study that used targeted deletion of *TSC1* in human embryonic stem cells. In two-dimensional NPC cultures and hCOs, only biallelic inactivation of *TSC1* was sufficient to activate mTORC1, resulting in dysplastic neurons and giant cells similar to those seen in TSC patients (Blair et al., 2018). The formation of TSC-like abnormalities in the brains of the Eker rat, a spontaneous *Tsc2* haploinsufficiency model, upon early postnatal irradiation to induce mutations, also supports the second hit model (Wenzel et al., 2004). Second hits in *TSC* patients have been consistently identified in SENs/SEGAs but not in tubers (Henske et al., 1996, 1997; Sepp et al., 1996; Wolf et al., 1997; Niida et al., 2001; Chan et al., 2004; Bongaarts et al., 2017; Giannikou et al., 2021). Moreover, a study using next-generation whole-exome sequencing identified second hit somatic mutations in 65% of SENs/SEGAs and 35% of cortical tubers from TSC patients (Martin et al., 2017), suggesting that additional mechanisms drive the formation of some tubers in TSC. However, most studies of tubers have analyzed tissue homogenate rather than single cells, an approach that supports the second hit hypothesis for tuber formation (Crino et al., 2010). To summarize, hiPSC, animal models and patient tissue analyses suggest a complex picture where heterozygous *Tsc1/2* mutations are sufficient to cause some neurological alterations perhaps at later stages, while initiation and early development of tubers and SEN/SEGAs may require two hits.

A new mechanism explaining the development of cortical lesions in TSC was suggested by a recent study which proposes that neurodevelopmental characteristics specific to humans, rather than a second hit in *TSC1/2*, might be the origin of some TSC brain lesions. Eichmüller et al. (2022) generated hCOs from 2 TSC patients with *Tsc2* germline mutations. The organoids developed lesions resembling cortical tubers or SENs/SEGAs in the presence and even in the absence of a second hit, as most tumors observed were heterozygous. scRNA-seq and histological analyses of the hCOs revealed a population of IPCs that gave rise to the giant cells that constitute tumors and tubers. Interestingly, these so-called caudal late interneuron progenitor (CLIP) cells were found to have intrinsically low levels of TSC1 and TSC2 proteins. These cells might therefore be particularly vulnerable even to germline heterozygous loss-of-function *Tsc1/2* mutations, which could be sufficient to cause them to develop into TSC lesions. Additionally, CLIP cells derive from the caudal ganglionic eminence (CGE), and CGE interneurons contribute to brain development in much greater proportions in humans than in mice (Hansen et al., 2013; Hodge et al., 2019). Thus, the intrinsic vulnerability of human-enriched neural progenitor populations, such as CLIP cells, to mTORC1 hyperactivation might explain the absence of detectable second-hit events in some cortical tubers and SENs/SEGAs in TSC, and may also contribute to the development of cortical lesions in other mTORopathies (Figure 2). However, as this study derived hCOs from 2 patients and second hit mutations may have been missed, replication and further validation are needed to support the CLIP cell model in TSC. *In vitro* organoid models also fail to replicate the neurodevelopmental and cell type complexity of the human brain that may contribute to the formation of tubers and SENs/SEGAs.

Heterozygous somatic gain-of-function mTOR pathway mutations can be sufficient to cause MCDs such as focal cortical dysplasia (FCD), HMEG or dysplastic megalencephaly (DMEG) (Lee et al., 2012; D’Gama et al., 2015; Mirzaa et al., 2016; Tee et al., 2016). Recent work suggests that the temporal window during which the mTOR pathway mutation occurs during development could influence the severity of MCD and seizure phenotypes (Figure 2). Pirozzi et al. (2022) used ultra-sensitive droplet digital polymerase chain reaction (ddPCR) to quantify common PI3K/AKT/mTOR pathway variants in surgically resected brain tissue from epilepsy and MCD patients. Consistent with prior studies, brain tissue from FCD patients had average lower variant allele fraction (VAF), indicating a later onset of somatic mutations, than tissue from patients with the more severe MCD syndromes HMEG or DMEG, in which the lesion encompasses more of the brain (Marsan and Baulac, 2018; Pirozzi et al., 2022). Interestingly, all children with early onset epilepsy had an average VAF > 5%, whereas those with VAFs < 1% had later onset epilepsy. This innovative study provides strong evidence for the hypothesis that the developmental stage at which somatic mTOR pathway mutations occur determines the degree of severity of patients’ morphological and neurological symptoms. This could explain some of the phenotypic heterogeneity between and within mTORopathies, whether they are caused purely by somatic mutations, or by a combination of germline and somatic events (Figure 2). Moreover, ultra-sensitive variant detection of somatic mutations in mTORopathies may become crucial diagnosis and prognosis tools which, provided that patients’ VAF is high enough, can be successfully performed not only from surgically resected tissue but also using saliva (Pirozzi et al., 2022).

## Discussion

Invertebrate and rodent models have been instrumental in uncovering roles of mTOR signaling in nervous system development. However, they do not allow us to fully understand mTOR signaling in human neurodevelopment and mTORopathies. hiPSC and hCO technologies have provided powerful new tools to characterize normal human neurodevelopment and model disease relevant processes. scRNA-seq, used to analyze the single-cell transcriptome of human fetal brain tissue and hCOs, provided the first evidence that mTOR signaling is active in oRG cells in the OSVZ. Subsequent studies using hCOs have confirmed the importance of the mTOR pathway in oRG and revealed new cell types and mechanisms that contribute to the neurological manifestations of mTORopathies.

Research in the last few years has greatly enhanced our understanding of the role of mTOR signaling in human nervous system development. However, the cellular and molecular mechanisms that precisely integrate mTOR pathway activity during the rapidly changing environment of the developing nervous system are still far from understood. The recent studies showing the relevance of mTOR signaling in oRG and CLIP cells suggest that there may be other, potentially transient, neurogenic cell types that utilize this pathway. Moreover, we know that in cultured cells, in addition to well-characterized substrates such as S6 kinase and eukaryotic translation initiation factor 4E (eIF4E)-binding protein (4E-BP), mTORC1 may have over 100 putative novel targets (Hsu et al., 2011; Yu et al., 2011; Robitaille et al., 2013). Therefore, as suggested by studies in *Drosophila* and mice (Avet-Rochex et al., 2014; Bateman, 2015; Maierbrugger et al., 2020; Vinsland et al., 2021), is mTORC1 target specificity important for the spatiotemporal regulation of neurogenesis? Identification of novel mTORC1 substrates in human developing cortical tissue or hCOs could provide answers to this question.

Recent discoveries made using hCOs combined with single-cell transcriptomics and the application of ultra-sensitive detection of somatic mTOR pathway variants from patient tissue may also have translational potential. mTOR pathway inhibitor drugs are currently used to treat the neurological manifestations of TSC (French et al., 2016; Stockinger et al., 2021). However, rapamycin and related drugs are only partially effective and so new treatments are needed, particularly for drug refractory epilepsy in mTORopathies. In future, understanding the role of mTOR signaling in human neurodevelopment may lead to therapeutic benefits for TSC and other mTORopathy patients.

## Author contributions

JB conceived the idea for the article. MG and JB wrote and edited the article. SU edited the article. All authors contributed to the article and approved the submitted version.
